# How People Use Web-Based Parenting Information to Support Others in Their Social Circle: Qualitative Descriptive Study

**DOI:** 10.2196/40043

**Published:** 2023-04-28

**Authors:** Reem El Sherif, Pierre Pluye, Virginie Paquet, Fidelia Ibekwe, Roland Grad

**Affiliations:** 1 Department of Family Medicine McGill University Montreal, QC Canada; 2 Health Sciences Library Université de Montréal Montreal, QC Canada; 3 School of Journalism & Communication Aix-Marseille University Marseille France

**Keywords:** consumer health information, information seeking behavior, child development, child health, information outcomes, health information, digital health, parenting, web-based information

## Abstract

**Background:**

Almost two-thirds of the North American population have searched for health information on the web, and the majority report searching on behalf of someone else in their social circle, a phenomenon referred to as *proxy seeking*. Little is known about how proxy seekers use web-based health information and the outcomes they experience.

**Objective:**

The main aim of this study was to explore why proxy seekers used a parenting website on behalf of parents in their social circle and the outcomes they reported.

**Methods:**

A qualitative descriptive study was conducted in the context of a partnership with a web-based parenting resource to explore the contexts and motivations for proxy web-based health information seeking, use of information, and subsequent outcomes. A total of 14 participants who self-identified as family members, friends of parents of young children, or professionals who worked with young children were interviewed, and a thematic analysis was conducted.

**Results:**

The following 4 reasons for proxy seeking were uncovered: for reassurance, out of personal curiosity, as part of a professional role, or following an explicit request from the parents. Information was used to provide informational support for parents or material support for a child. Positive outcomes of using the information and some of the resulting interpersonal tensions were described.

**Conclusions:**

This study provides an in-depth look at proxy seeking behavior and outcomes among users of a web-based parenting resource.

## Introduction

### Online Health Information

In 2020, over two-thirds of Canadians (69%) reported searching for health information on the web [1]. This is in line with results from the Health Information National Trends Survey in the United States between 2008 and 2017, in which two-thirds of respondents reported turning to the internet first for health information [2]. *Online health information* (OHI) is the term generally used to refer to information on all aspects of health (including mental, physical, and social) created for and directed toward the general public [3]. OHI is available in many formats, such as text and video, and is available at government health sites and from professional organizations, health journals, and blogs among other sources. Moreover, individuals are exposed to OHI “posts” shared by their network through social media platforms such as Facebook [4].

### Outcomes of OHI Seeking

Previous work has explored the different outcomes of OHI seeking from the individual self-seeker’s perspective, and these outcomes are described at 4 levels: situational relevance, cognitive impact, use, and subsequent health outcomes of information [5]. People can use OHI in many ways, most commonly to discuss with health care providers, engage in their own health care, modify or comply with a management plan, or support relatives or friends with health conditions [5]. Using OHI is generally associated with positive perceived outcomes such as increased empowerment of consumers and their families and improved health outcomes [6-9]. There may be negative outcomes (referred to as *tensions* in previous work), such as increased anxiety or worsening of the patient-physician relationship; however, there are strategies, such as providing trustworthy resources, to reduce these tensions [10].

Several contextual factors are associated with OHI outcomes. These include age, education, income, eHealth literacy, and sources of social support [5]. Source of social support is an important factor because one of the main reasons people search for and use OHI is to support their relatives or friends with health conditions [11]. Moreover, findings from a study exploring internet use trends between 2008 and 2013 showed a significant increase in the involvement of family and friends to obtain health information [12]. Individuals are sometimes more likely to turn to their social circle to make sense of the information they find, rather than discuss it with a health professional [13,14].

### Social Support

Social support is one of the positive products of “social relationships,” which may have short- and long-term effects on health, for better and for worse, depending on their quality and quantity [15]. A model by Uchino [16] describes 2 broad dimensions of support: structure and function. Structural aspects of support are the extent or composition of one’s social network (size, contact, type, density, and strength) and the interconnections among them. Functions are organized along 2 levels—perceived support and actual support—and have 3 aspects that are highly related to each other—informational, emotional, and tangible aspects. Most relevant to this study is informational support, which includes the provision of advice or guidance, and it may provide direction and carry an emotional message when received from a close source. Informational support can be construed as supportive, unsupportive, or mixed depending on context [17-19]. Emotional support is the offering of warmth and nurturance, including encouragement, empathy, trust, affection, and other positive facets, that can reduce stress or other negative emotions [16,20]. Tangible support involves the provision of material (practical) aid [16,20].

Informational social support can occur in 2 ways: an individual can request informational support from the social support provider (by discussing health information with them and asking for their help) or it can be unsolicited (the provider searches on behalf of the individuals and shares it with them). In the first case, for example, an individual’s selection of the source of information depends on the individual’s needs and expectations, so they may consult their friends and families when they need “more tailored emotional support in obtaining complex and serious health information” [21,22]. In the second case, a social support provider is aware of the individual’s information need (eg, recently diagnosed health condition) and searches for information on their behalf to share with them, supporting their health care management. Although informational support has been explored in the past, few studies have focused on its outcomes in an OHI context, and none have looked at it from the perspective of both the provider and the receiver.

### Proxy OHI Seeking

Proxy information seekers can be defined as “those who seek information in a nonprofessional or informal capacity on behalf (or because) of others without necessarily being asked to do so” [14]. Proxy seekers may also be “experts,” such as health librarians or health care professionals with specialized knowledge or skills to use the information with the individual with whom they share a personal relationship [23]. Although this phenomenon of proxy information-seeking behavior has been explored in the literature, especially in relation to health information, few studies have explored the context of proxy OHI seeking being linked to the use of OHI and subsequent health outcomes.

This constitutes a critical knowledge gap. People may be able to overcome low eHealth literacy by discussing the information they find with others [10]. Proxy seekers in a person’s social circle may help them overcome information-seeking barriers and illness challenges (eg, they are too physically weak or mentally incapacitated to search themselves) [14]. By better understanding how proxy seekers use information with people in their social circles, information providers can better adapt the information to meet their needs, and public health interventions can target patients’ friends and family with information for dissemination and use [24]. Thus, the objective of this qualitative study is to explore the motivations, contexts, and outcomes of proxy seeking behavior from the perspective of proxy seekers.

## Methods

### Theoretical Model

**Figure 1:**
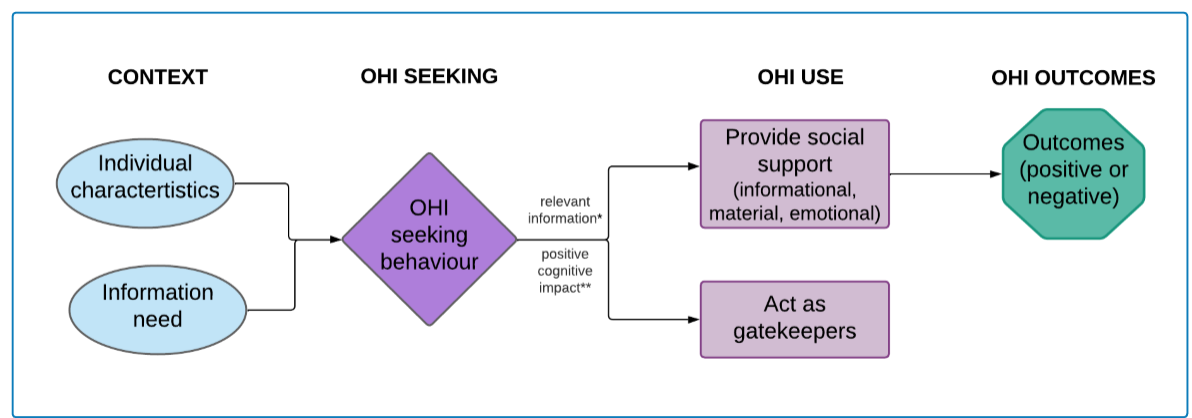
Outcomes of proxy online health information (OHI) seeking framework. *If no relevant information is found then there is no OHI use, as well as no outcomes **If there is a negative cognitive impact then there is no OHI use as well as no outcomes.

The model guiding this work was developed by following a mixed studies literature review on proxy OHI-seeking behavior and was published elsewhere [25]. The findings from the thematic analysis of 28 included studies were used to revise the existing conceptual framework [5]. Our Outcomes of Proxy OHI Seeking model is presented in (Figure 1). Individual characteristics such as age and gender influence proxy OHI seeking, for example, most studies report that proxy seekers are more likely to be women and aged between 31 and 64 years [13,26]. The OHI-seeking process is triggered by another individual’s information need, which may be explicit (stated to the proxy seeker) or implicit (eg, observed by the proxy seeker). The proxy seeker will then actively search for or passively monitor OHI to fulfill this information need. When they find a situationally relevant information object that has a positive cognitive impact, they will use it to provide informational, tangible, or emotional support for someone else. A common example of use would be the sharing of information between caregiver and patient either directly by sending them a link or printout or indirectly by discussing the information found. The proxy seeker could also act as information gatekeepers for the individual to reduce the burden of information overload or prevent conflict. OHI use will lead to separate outcomes experienced by the individual and the proxy seeker. These outcomes are generally positive; for example, information can help people make a health behavior change like quitting smoking or allow them to feel more confident and able to discuss the information with their health care providers and request different management options. There are also negative outcomes such as increased anxiety for both the proxy seeker and the individual, for example, with conflicting information or with conflicting preferences for information in general [25].

### Resource: Naître et Grandir

The Naître et Grandir (N&G) website provides free, expert-based, web-based parenting information content in French that caters to people with lower health literacy levels (Grade 8 reading levels) with additional audio and video content [9]. Web-based parenting information, which encompasses all mental, physical, and social aspects of children’s health, is a large subset of web-based health information on the internet [27]. In addition to directly accessing the website, N&G readers can sign up to receive a weekly newsletter containing parenting tips and links to N&G webpages tailored to their child’s age and evolution.

N&G is funded by the “Lucie and André Chagnon” Foundation, a Quebec-based philanthropic organization that seeks to create conditions and environments that are favorable to the educational success of children. Since 2014, the research team of 3 coauthors (PP, RG, and RES) has worked in partnership to implement the Information Assessment Method (IAM) questionnaire to evaluate this parenting information. When N&G readers land on a web page corresponding to a specific topic (directly or from the newsletter link), a lateral tab appears, inviting them to complete the survey. The first question asks the respondents to identify with one role for the purpose of this specific web page they are rating: parent, grandparent, family member, friend or neighbor, or professional who works with children aged 0 to 8. N&G editors have been able to improve their informational content using the comments provided by the readers through the IAM questionnaire [28]. Further details on the IAM and quantitative analysis of responses from parents and entourage members have been published elsewhere [9]. The translated version of the current questionnaire is available elsewhere [29].

### Study Design

A qualitative descriptive study was conducted using semistructured remote interviews with IAM respondents who identified as entourage members. This type of study is used to provide an accurate account of the events or experiences of participants attributed to those events [30].

### Ethics Approval

Institutional Review Board approval from McGill University was obtained before the start of the study (Institutional Review Board study number: A12-B73-18A). Methods and results were reported using the COREQ (Consolidated Criteria for Reporting Qualitative Research) [31]. This study was the second component of a mixed methods convergent study, and the details of the quantitative study have been published elsewhere [29].

### Study Participants

A purposive sampling strategy was used to select potential participants from a data set of IAM questionnaires received between April 13, 2019, and March 30, 2021. IAM responses that were completed by an entourage member who agreed to be contacted for an interview were exported into a separate Excel (Microsoft Corporation) file. After excluding those with no valid email addresses, the final list included 71 potential participants (25 grandparents, 17 family members, 15 friends or neighbors, and 14 professionals caring for children). An invitation email was sent to these potential participants, 4 per week, in the order they had completed the questionnaire, from the oldest to most recent.

### Data Collection

An interview guide was developed using an iterative process based on the Outcomes of Proxy OHI Seeking model. The guide was pilot-tested with 2 graduate students, and the researcher’s notes and interviewee’s feedback were used to revise it and produce the final version. A total of 14 individual semistructured interviews were conducted in French over the phone or videoconference (Zoom [Zoom Video Communications Inc]), depending on each participant’s preference. When participants responded to the invitation email, they were sent the consent form and were asked to respond with their written consent and any questions that they had.

After being introduced to the purpose of the study, the participants were asked general questions regarding web-based consumer health information and the context and resources of their information-seeking behavior. They were asked about their role as entourage members and about the members of their social circle with whom they were frequently in contact. They were reminded of the N&G web page they had rated using the IAM questionnaire and were asked to describe how and why they had landed on that page. Finally, they were asked how they used the information on the page and what outcomes they perceived as a result. The interviews were recorded, and the recordings were transcribed by a professional transcriber, translated into English by 2 of the authors, and analyzed using web-based translation software (deepl [32]).

### Data Analysis

Transcripts were imported into NVivo (Release 1.5; QSR International), and a deductive-inductive analytical approach was adopted for coding [33,34]. A coding manual was created and discussed with another coauthor (VP). The codes were progressively clustered into themes and subthemes. Coding was conducted by the first author by participant and by coding meaningful extracts into the major themes first; then, the extracts in each theme were coded into subthemes. Themes and subthemes were discussed with the coauthors throughout the coding process, and their feedback was incorporated into the coding manual.

**Figure 2:**
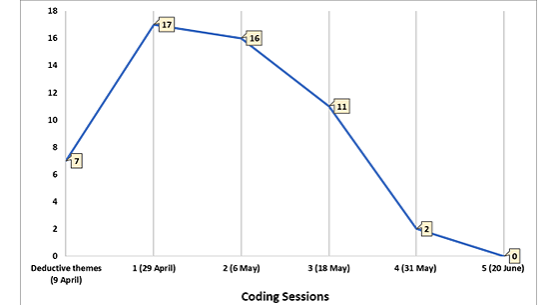
Qualitative data analysis: saturation of themes reached after 4 coding sessions.

The interview transcripts were analyzed over 5 coding sessions, as shown in Figure 2. During the fourth coding session (after 12 interviews had been conducted and analyzed), only 2 new themes emerged. Two more interviews were conducted and analyzed, and no new themes emerged. Therefore, saturation had been reached, and data collection stopped.

## Results

### Description of Participants

In total, 14 participants were interviewed, comprising 5 (36%) grandmothers, 4 (29%) family members, 4 (29%) professionals, and 1 (7%) friend. Most of them were female (12/14, 86%) and had a bachelor’s degree or higher (8/14, 57%). Respondents completed an average of 4 IAM questionnaires over the 2 years of study period (range 1-14). Full details of the participants are presented in Table 1.

**Table 1:** Participant characteristics.

Pseudonym	Age group (years)	Income^a^ (CAD $)	Education	Profession	Entourage type	Average internet use (hours/day)
Alisson	26-44	>60,000	Bachelor’s	Teacher	Family	2
Sarah	26-44	<60,000	High-school diploma	Retailer	Family	5-6
Mark	26-44	<60,000	College	Practical technician	Family	3
David	>45	<20,000	High-school diploma	Unemployed	Friend	2-3
Mary	>45	>60,000	College	Admin in adult education center	Grandmother	3
Nadia	>45	>60,000	Master’s	Research coordinator on aging	Grandmother	3
Sophie	>45	N/A^b^	Bachelor’s	Spanish interpreter	Grandmother	5
Nathalie	>45	>60,000	Master’s	Retired	Grandmother	2-3
Joelle	>45	>60,000	Master’s	Retired school principal	Grandmother or professional	3
Florence	26-44	>60,000	High-school diploma	Kinder garden child educator	Mother or professional	1-2
Norma	26-44	>60,000	Bachelor’s	Nurse	Professional	4
Alice	26-44	>60,000	Master’s	Psychoeducator (0-7 years old)	Professional	3-4
Emilia	26-44	<60,000	Certificate	Kindergarten educator	Professional or friend	>8 (work + personal)
Mathilde	<25	<60,000	CEGEP^c^	Student	Sister	1

^a^The Institute for Research and Socioeconomic Information has identified CAD $60,000 (CAD $1=US $0.70) as the sustainable income index for a family of 4, indicating the income a household should have to not only meet its basic needs but also get out of poverty, while the index for a single person is approximately CAD $27,000 [35].

^b^N/A: not applicable.

^c^CEGEP: CollèGe D'Enseignement GéNéRal Et Professionnel. It is the equivalent of Grade 13 in Quebec, Canada.

### Contexts and Motivations of Proxy OHI-Seeking Behavior

Two main themes were discussed in relation to the context of proxy information-seeking behavior: individual characteristics of the entourage members and the information needs that triggered the seeking of web-based parenting information. The entourage members were reminded of the N&G web page they had landed on before completing the IAM questionnaire and were prompted to recall the reason they were on that topic. The specific N&G web pages and the reasons are reported in Table 2.

All the participants described who they considered as their social circle, and in addition to family members and friends, some professionals included their colleagues and clients (parents of children in their care) in their social circle. All entourage members were in close contact with the people for whom they were seeking information by proxy. This contact may be in person, but many also described remote contact because of either geographic location or restrictions imposed by the pandemic:

Let’s say they don’t live that far away, but with the COVID context, what I was doing, I was Face Timing with them on the weekends, because, among other things, their mother was extremely strict about visitation and all that. But let’s just say in a context, if I look at past years, we would see each other almost every week, we would go for a little walk, but that hasn’t been the case since March 2020.

Nathalie, a grandmother

Proxy information seeking was triggered by different motivations falling under 4 broad themes: for reassurance, out of personal curiosity, for work as a caregiver, and following an explicit request from someone else. Excerpts corresponding to each theme are presented in Table 3. Several entourage members described wearing multiple hats, as professionals who worked with children and as family members or friends with children in their personal circle.

**Table 2:** Latest Naître et Grandir (N&G) web page rated by the participants.

Participant	Last N&G page rated	Context
Alisson (family)	Development: Around 5 years old	“It was really from the beginning of [my nephew’s] life, when he was very small, because he came into the world prematurely and he had some pretty close follow-ups in the first months of his life.”
Sarah (family)	Learning to walk	(Could not recall the specific N&G page so referenced another page). Nephew: “Well, when he first started teething, I was wondering if it was normal, say, for him to have a lot of fever, rashes, things like that, what to do to help with the toothache*.”*
Mark (family)	Verbal dyspraxia	“Yes, it was about my son’s behavioural problems...It was one of the few times that it was pretty clear that I was overwhelmed by the situation. The calls to the family didn’t inform me well enough, in my opinion, about the situation, which was still pretty sharp and pretty specific, so I went looking for very specific information on a specialized and credible site that I knew and came straight to it.”
David (friend)	Tantrums: Understanding them to better intervene	Friend’s child: “This is not the first time I’ve seen a child have a meltdown. It was because she was coming up to three years old and I was wondering what the age range really is in that.”
Mary (grandmother)	The benefits of music	“My interest in the education of this grandson.”
Nadia (grandmother)	The benefits of reading with your child	“Granddaughter of a child who is one and a half years old...She comes to spend, usually, one day a week on weekends at my house*.”*
Sophie (grandmother)	2 to 2.5 years: intellectual development	Grandchild: “How to understand her, but also how to interact well so that I can give her all the...so that her development is as good as possible.”
Nathalie (grandmother)	The child who doesn’t like kisses	“With the [grand]children I live with now, they have two completely different personalities. Bella, the little one, she is extremely affectionate. She always wants to be stuck to us. Matteo is the complete opposite. He’s a very independent child, who has to be approached gently, and me, anyway, I don’t want to impose my kisses and all that.”
Joelle (grandmother and professional)	Grief in children	“It was my daughter-in-law who passed away...So, I shared that information first with my son and his girlfriend. I sent them the link...The child lives with them full time now. I sent him the link to Naitre et Grandir to encourage him to go see it...”
Florence (mother or professional)	The child who doesn't talk yet	“I have my own private home daycare...As far as my son or my friends’ children are concerned, because we talk about it a lot, or the kids I currently have in my daycare, because we are confronted with little viruses, little bacteria, and big worries from parents as well, Naître et grandir is a great, great source.”
Norma (professional)	The basics of breastfeeding	“I’m a nurse. I work in early childhood. I’ve always worked in the childcare setting.”
Alice (professional)	Sleep: effects on development and behavior	“I am a psychoeducator for young children aged 0-7. My clientele is mostly children with autism spectrum disorders and their families as well. Yes, it was for one of my families that I’m following up with.”
Emilia (professional or friend)	Yogurt: Which one to choose? & Food rewards	“It’s because basically in a course where I’m going to be doing observations, there’s also the health element, and I talk to students sometimes about nutrition and being able to offer a variety without necessarily threatening to take the dessert away.”
Mathilde (sister)	Lessons and homework: accompanying your child	“Sometimes, also, on health, it’s more my little brother. But for kids in general, it’s mostly for my babysitting.”

**Table 3:** Themes related to motivations for proxy information seeking (N=14).

Theme	Excerpt	Frequency, n (%)
For reassurance	“I was clearly overwhelmed by the situation. It was one of the few times that it was pretty clear that I was overwhelmed by the situation. The calls to the family didn’t inform me well enough, in my opinion, about the situation, which was still pretty sharp and pretty specific, so I went looking for very specific information.” [Mark, a family member]	4 (29)
Out of personal curiosity	“It’s more of a special interest, because now I’m a grandmother and the context is that I don’t have a spouse anymore, so my priority now is my children and my grandchildren.” [Nathalie, a grandmother]	3 (21)
For work as a caregiver	“It was to go and get ideas for games to incorporate into my program, because I was going to explain something...Learning, active play, we explain that a little bit, and here I had to give examples of games” [Alisson, a family member who is also a teacher]	4 (29)
Following an explicit request	“Actually, it was to reassure a pregnant friend about COVID vaccine” [Norma, a professional]	1 (7)

### OHI-Seeking Behavior

Participants described their strategies for searching for OHI and how they assessed the credibility of the information they found. Many participants would typically start searching for OHI by entering ≥1 keyword into a search engine (eg, “Googling the word ‘vaccine’”) and clicking on the first few links or selecting links to resources they recognized. In contrast, 2 participants mentioned that they started from websites they had bookmarked, including N&G, rather than Google.

Participants had different ways of thinking about the credibility of a website, and for the most part, they preferred websites from institutions they recognized:

Mostly I look for it to be recognized, for it to be something I’ve heard of or seen before, if it’s a medical clinic I know, Mayo Clinic in the United States.

Alice, a professional

Some participants would check the credentials of the authors, and the validity of the references. Websites that had “too many ads” or “several spelling mistakes” were considered less credible. Several described using a critical attitude when assessing websites:

There’s a bit of intuition, there’s a bit of experience. I have a little trouble believing anything too. There’s a lot of quackery on the Internet, and I’m wary of sites that aren’t officially licensed.

Mary, a grandmother

After checking a few sites or trying different keywords, the seeker would decide that they had found something relevant after triangulating from different resources:

After three references that talk about the same thing, that give about the same result.

Alice, a professional

Some participants described the cognitive impact of the information, which gave them personal satisfaction to know more, allowed them to learn something new, or confirmed something they already knew:

It’s because of what I’ve already studied and what I know, and then I’m mostly looking for either validation of the information I already have or to see if it’s already out there in the mainstream at this point, if there is another way to explain it more easily.

Alice, a professional or aunt

### Using Relevant Web-Based Parenting Information

Participants described how they used the information they found on the N&G web page that they recently rated in symbolic and instrumental manners. Themes related to information use are listed in Table 4. With regard to *symbolic use*, most used the information to provide informational support to someone in their social circle. They sometimes shared the link to a relevant web page directly with the child’s parent:

A lot of times I’ll send her [the child’s mother] a little message on Facebook in a private message, I’ll send her the link outright.

Sophie, a grandmother

The 29% (4/14) of professionals described situations in which they would share the links to N&G web pages with the parents of children in their care after the parent had mentioned specific concerns on the topic:

I have a child who went to get vaccinated, and the mom was worried because he had had a reaction to his vaccines before and now, he was on the next vaccine. So, to reassure her, I sent the link two days ago to the mother which came from Naître et grandir.

Florence, a professional

Other times, participants discussed the content of the web page without sharing the link itself:

I share my perspective (with my son), but my perspective is kind of informed by that information from N&G .

Nadia, a grandmother

The entourage member would sometimes also discuss the information they found with people other than the individual for whom they were searching to help them make sense of it:

I am lucky enough to work with professionals in speech therapy, special education, and psychology, so at work it’s fun to have a credible second opinion, to confirm or to refute.

Mark, a family member

In contrast, in some situations, they did not share the information at all, often to avoid tension or conflict with the individual.

For example:

I’ll take on the role of the specialist with respect to my sister, so sometimes that leads to discussions that are less pleasant.

Emilia, a professional

Overall, 14% (2/14) of grandmothers discussed not sharing the information because they did not want to appear too intrusive or too judgmental about their children’s parenting, as one of them said the following:

Giving out information that is not sought after is, in my opinion, a waste of time

Nathalie, a grandmother

Another way the participants used the information was to provide material support (ie, *instrumental use*). This was specifically true for family members who were occasionally entrusted with the care of a child. Mathilde described using the information she found to help her brother with his homework while she was babysitting him in the evenings. A total of 36% (4/11) of grandmothers described learning new ways to interact with their grandchildren while they were spending time with them:

I’m going to make him do a recipe. We’re not going to do math, we’re not going to do written problems, we’re going to do a muffin recipe.

Alisson

Finally, one grandmother described providing emotional support to her bereaved son after she read relevant information on N&G:

It was more with my son that I talked about it, but really, him, it wasn’t so much about where I found the information as it was about discussing the grief.

Joelle, a grandmother and professional

**Table 4:** Themes related to proxy information use (N=14).

Theme	Excerpt	Frequency, n (%)
Did not share with someone	“I don’t want to give them the impression that I’m watching how they are. I find that everyone gives so much advice when you’re a parent. Everyone has their idea of what’s best and what not to do and all that, so I try to gauge that, not put too much on it. It’s more that I keep it in mind for if they ever bring it up or something like that.” [Nadia, grandmother]	7 (50)
Doing something	“It helped me to be able to guide my brother in his learning at school, to know how to help him more, what I should do.” [Mathilde]	5 (36)
Shared with someone else	“I shared that information first with my son and his girlfriend. I sent them the link. There are things that I photocopied and showed to my son.” [Joelle]	10 (71)
To discuss with HCPs^a^	“I am lucky enough to work with professionals in speech therapy, special education, and psychology, so at work it’s fun to have a credible second opinion, to confirm or specially to refute.” [Mark]	3 (21)
To discuss with others	“I usually print the page out or email it to the parents to read. It depends. Sometimes they read more when it’s paper because I email it and it gets lost with all the other emails. But I give, and afterwards, at my meeting after: ‘What did you understand? Did you get a chance to read it? Do we read it together?’ and so on.” [Alice]	5 (36)
To make decisions	“I’m going to go back and read it again to confirm, actually, that the approach that I want to implement is really in line with the information that I’ve had, because I wouldn’t want to go on and just like stay within my capabilities and it’s like just motivated me, but not really being applied.”[Mark]	1 (7)
To provide emotional support	“It was more with my son that I talked about it, but really, him, it wasn’t so much about where I found the information as it was about discussing the grief.” [Joelle]	1 (7)

^a^HCP: health care professional.

### Outcomes of Using Web-Based Parenting Information

Themes related to the outcomes of information use are presented in Table 5. The reported outcomes of using N&G information were generally positive. The most common outcome was improvement in the relationship with others. In the case of Sophie, reading the information on her granddaughter’s intellectual development allowed her to better understand her behavior. This allowed her to change her interactions with her granddaughter, which led to them being more comfortable with each other. Another grandmother, Nadia, explained how the information allowed her to be more reassuring and supportive of her son and daughter-in-law. After sharing information a few times and feeling validated, one grandmother described feeling more comfortable discussing what she had read with her son again in the future, and a professional described how sharing information with the parents of a child in her care led to better discussions.

Another commonly reported outcome was reassurance. Sarah, a family member, described feeling reassured after finding answers to her questions about miscarriages on the web. She discussed the information she had found with her partner, and they both felt reassured as a result. Norma, a professional, was approached by her pregnant friend who was concerned about the COVID-19 vaccine. After Norma shared the N&G web page on the safety of the vaccine during pregnancy, her friend was reassured and proceeded to keep her vaccination appointment.

Some participants also felt more confident in making decisions with others and being more involved in the care of the child as one grandmother described:

Yes, it gives me more confidence that I’m doing it the right way and that it’s okay to do it, let’s say. I guess it gives me more confidence in how I’m intervening with her

Nadia, a grandmother

One professional reported that the parents in her care were the ones who felt more confident in their interventions with their child following a discussion of the information she had shared:

Yeah, it’s not perfect, they don’t all change their behavior, because it’s still a loop, but they quietly start to realize, and then the kids’ behavior starts to decrease, and then the parents become more confident in their interventions.

Alice, a professional

A total of 2 (14%) of the 14 participants described negative outcomes or tensions as a result of sharing information. Alisson who shared information with her sister describes one such outcome:

I have to be careful, because she didn’t take it very well. She, she thought I was doubting her...she wasn’t too keen on me telling her about it after all.

Alisson

Emilia, who is a proxy seeker both as a professional and as an aunt, described how her sister would sometimes be resistant to the advice and information she shared:

At one point she told me he wasn’t that bad, but sometimes when she feels exhausted about it, she tells me about it like it’s a mountain, and other times, once I bring the information, it seems like she doesn’t want to.

Emilia, a professional

Emilia concluded that she had better experience of sharing information in a professional context than in a personal one.

**Table 5:** Themes related to outcomes of proxy information use (N=14).

Theme	Excerpt	Frequency, n (%)
Improved relationships	“Yes. In the relationship, it’s clearer when we talk. They already know what we’re talking about, how, and they know.” [Alice]	4 (29)
Less worried	“Not necessarily, but it reassured me. Going to see that information really reassured me. I was kind of full of questions and stuff and I was not sure about everything, so I was like, ‘Okay. At the same time, I do not necessarily want to call a doctor and ask him a little bit...probably bother him for nothing.’ When I saw that, I was like, ‘Okay, that’s good. Okay, that explains some things.’ It put some answers to my questions, and I was better with myself after reading that and I felt much better.” [Sarah]	3 (21)
More confident in decision-making	“Yes, it gives me more confidence that I’m doing it the right way and that it’s okay to do it, let’s say. I guess it gives me more confidence in how I’m intervening with her.” [Nadia]	3 (21)
Tension	“I have to be careful because she didn’t take it very well. She, she thought I was doubting her...I was curious, and at the same time I told her about it, but she wasn’t too keen on me telling her about it after all. ‘You don’t mind your own business, old girl.’” [Alisson]	2 (14)

## Discussion

### Principal Findings

This study explored the motivations, context, and outcomes of proxy seeking behavior from the perspective of 14 entourage members of parents of young children, seeking information on a web-based parenting resource. Most respondents played one or more roles as family members, friends, or professionals who worked with younger children. They were proxy seeking for reassurance, out of personal curiosity, as part of their professional role, or following an explicit request from their parents. They used the information to provide informational support (either by sharing the web page or discussing its content) or to provide material support for a child in their care. In some cases, they did not share the information to avoid causing tension with the parents in question. Furthermore, they generally reported positive outcomes of using the information: feeling less worried, finding an improvement in their relationship with the parent or child, and feeling more confident in future interactions. Some interpersonal tensions were described as a result of sharing the information, specifically when it was unsolicited and when it was shared in the context of a personal relationship.

This study highlights the role of social support in web-based health information–seeking outcomes. Social support has consistently been linked to better health [16,36,37]. Several explanations have been proposed to explain why this occurs; for example, social support can act to reduce the impact of stress, which subsequently improves mental health [15]. Another potential explanation is that the provision of informational support encourages the receivers to manage their health, as demonstrated in a study that explored the relationship between maintaining an improved cardiovascular health status and social support networks [38]. If we use pregnant women as another example, those who were more satisfied with perceived and received social support initiated prenatal care earlier than those who were less satisfied [39]. Pregnant women who received more informational support from people in their social network delivered newborn infants with higher APGAR (Appearance, Pulse, Grimace, Activity, and Respiration) scores (a measure of health 5 minutes after birth) and higher birth weight [39,40]. Although informational support has been explored in the past, few studies have focused on its outcomes in the OHI context.

Negative outcomes were reported by 2 participants after proxy OHI use, specifically related to interpersonal tension. In general, negative outcomes are rarely reported: a literature review found limited reports of patient anxiety or decisions to refuse cancer treatment [41]. There were 2 studies that reported that the proxy seekers themselves experienced more anxiety sometimes owing to information overload [42,43]. The proxy seeker and the individual did not always have the same approach to OHI; situations in which the individual did not want to “know” or ignored the information led to tension and conflict [44,45]. Moreover, a mixed methods study in the context of patients with diabetes reported that the greater the proxy OHI seeking, the lesser the family members were perceived to be supportive, owing to attempted influence and interference by proxy seekers [46].

To our knowledge, this is the first qualitative study to focus on the entourage of young children’s parents in the context of web-based parenting information. A recent review of the literature conducted by the authors on proxy OHI-seeking behavior included 10 qualitative studies: 6 explored the perspectives of both proxy seekers and self-seekers; 3 explored the perspective of proxy seekers only; and 1 explored the perspective of self-seekers who relied on others to make sense of the information. Most studies (7/10, 70%) focused exclusively on caregivers of patients diagnosed with a chronic or acute illness; 2 focused on the care of older adult family members; and only 1 explored the health information–seeking behavior in the general population. The latter explored how Singaporeans came to make sense of web-based health information seeking and described how people’s roles within family relationships necessitated proxy seeking [47]. Similar to our study, that study reported positive outcomes of proxy OHI seeking and use, such as feeling less worried.

In this study, most participants were grandparents, who also represented 12% (6309/51,325) of N&G-IAM survey respondents in the previous quantitative study [29]. One contribution of this study is the perspective of older OHI consumers as the proxy seekers rather than the recipients of support. In 2018, almost 71% of Canadians aged ≥65 years used the internet, and in 2020, almost 50% searched for health information on the web (Statistics Canada [48]) [1]. The grandparents in our study were frequent internet users who used the information they found on the web to provide informational and material support to their children and grandchildren and reported benefits such as improved relationships and increased confidence in their abilities. A recent study that explored web-based health information seeking in older adults reported that self-seeking and proxy seeking were active coping strategies to reduce health risks and improve health promotion in health care [49]. A large number of N&G readers are professionals who work with young children outside the health care field, as reflected in our study population that included 5 educators. Although they are considered important influences in the lives of young children, few studies have explored the OHI-seeking behavior of these professionals, as reported in a recent systematic review [50].

Another strength of our work is the partnership with N&G. One major limitation of empirical studies on OHI is the inability to assess the quality of the OHI used by the participants. N&G is an expert-based OHI source for people with low health literacy levels, with additional audio and video content [9]. By decreasing the health literacy gap, people are better able to process and use information [51]. This provides a context in which the phenomenon of proxy OHI seeking can be explored without major concerns about the quality of the information. N&G is neither a traditional scientific and medical resource nor a blog. In previous research, comments from the readers of websites and blogs have been analyzed, but few researchers have conducted interviews with users of parenting websites to explore their motivations and outcomes in-depth [52].

Our study allowed us to explore different concepts within the Outcomes of Proxy OHI Seeking model in the context of entourage members of parents of young children [25]. The context, OHI-seeking behavior, OHI use, and outcomes described in this study provide tangible examples to illustrate the different outcomes. Therefore, this work provides empirical support for the Outcomes of Proxy OHI Seeking model. In addition, we can now improve the IAM questionnaire to allow for response items catered to entourage members, as the IAM was originally developed and validated with parents.

There are 4 main limitations to our study. Although we attempted to recruit more men, most participants were women (12/14, 86%), and this corresponds to the gender of the respondents to the IAM questionnaire (on average, 90% of respondents were women). Although this lack of heterogeneity may be considered a limitation, studies have consistently reported that most OHI proxy seekers are women, as reflected in our sample [13,53-55]. In a recent analysis of IAM responses by N&G users with low socioeconomic status, our team reported that fathers were more likely to report the benefits of N&G information than mothers [56]. This highlights the need to target men OHI seekers with inclusive information and to explore their use of OHI in future studies. The second limitation is that we only explored the viewpoint of proxy seekers and did not interview the parents for whom they were searching. These interviews may have provided a fuller picture of this phenomenon but were beyond the scope of this study. The third limitation is that the author who conducted the qualitative data analysis (in English) was not the author who conducted the interviews (in French). To mitigate this, the authors held frequent meetings throughout the study: before and after each interview and during the qualitative data analysis. The final limitation is that the contextual factors related to proxy OHI-seeking outcomes were not assessed within the scope of this study. Future work can explore the relationship between proxy seekers’ characteristics and the outcomes they report.

### Conclusions

This study supported our Outcomes of Proxy OHI Seeking model. We plan to use this study to improve the IAM questionnaire implemented by information providers in Canada. From a practical standpoint, this is an important topic for information specialists, primary health care practitioners, and public health officials. By better understanding how an individual’s entourage uses information and experiences subsequent outcomes, information providers can better adapt their information to meet their needs, while health care practitioners can target the patients’ entourage with web-based health information resources. Public health interventions aimed at supporting parents can do so by improving their social networks (eg, by facilitating longitudinal relationships with proxies such as other parents or extended family members). Other professionals involved in the support of parents and their children (eg, day care educators and teachers) can be specifically targeted with reliable OHI to promote positive outcomes.

## Abbreviations

APGAR: Appearance, Pulse, Grimace, Activity, and Respiration
COREQ: Consolidated Criteria for Reporting Qualitative Research
IAM: Information Assessment Method
N&G: Naître et Grandir
OHI: online health information
